# Epidemiological study of hantavirus in Southern Brazil, 2009-2019

**DOI:** 10.1590/S1678-9946202668028

**Published:** 2026-04-10

**Authors:** Felipe Manoel Gimenez de Oliveira, Paulo Augusto Esteves, Ricardo Evandro Mendes

**Affiliations:** 1 Instituto Federal Catarinense, Programa de Pós-Graduação em Produção e Sanidade Animal, Concórdia, Santa Catarina, Brazil; 2 Embrapa Suínos e Aves, Concórdia, Santa Catarina, Brazil; 3 University of Georgia, College of Veterinary Medicine, Department of Pathology, Veterinary Diagnostic Laboratory, Athens, Georgia, USA

**Keywords:** Public-health, Risk-based surveillance, Hantavirus infections, Viral zoonoses

## Abstract

Brazil has the highest number of hantavirus cardiopulmonary syndrome cases on the American continent, with Santa Catarina being the state with the most notifications. This retrospective longitudinal study aimed to describe the epidemiological profile of 177 hantavirus cases reported in Santa Catarina from 2009 to 2019, using data from the Notifiable Diseases Information System (SINAN). Statistical analyses of socio-demographic, clinical, and epidemiological data revealed that the typical patient was a male of working age with low educational attainment, living in a rural area. The highest incidence occurred in the Santa Catarina West, Midwest, and Mountain regions, strongly associated with agricultural activities. The case fatality rate (CFR) was highest among the 15-19-year age group. Clinical risk factors for death included respiratory signs, increased hematocrit, and the need for mechanical ventilation. Patients who sought early care had a higher CFR, possibly due to the initial difficulty of differentiating hantavirus from other viral diseases. Conversely, regions with higher notification rates showed lower CFRs, suggesting better surveillance. This study highlights critical areas for public health intervention and the key characteristics of hantavirus patients (males in rural areas and adolescents aged 15-19 years in regions with low notification rates) and areas for public health intervention. Training for medical professionals in regions with low notification rates should aim to reduce lethality, especially in regions with low reported cases (Itajai river delta and South), where underreporting may be occurring. Furthermore, the high lethality in adolescents and in patients with non-specific initial symptoms requires greater awareness. This study shows the utility of a governmental database in identifying epidemiological patterns and creating public health strategies tailored to regional specificities.

## Introduction

Hantavirus is an acute febrile zoonotic illness with two distinct clinical forms: hemorrhagic fever with renal syndrome (HFRS), prevalent in Europe and Asia, and hantavirus cardiopulmonary syndrome (HCPS), which is endemic to the Americas^1^. Hantavirus disease is a multispecies infection caused by several viruses within the genus *Orthohantavirus*, each associated with specific rodent reservoirs and distinct clinical outcomes^1^. Transmission occurs primarily by inhalation of aerosolized rodent excreta. However, regions such as Europe, Asia, and South America^2^ have documented rare cases linked to contaminated food, rodent bites, and even person-to-person transmission. Wild rodents serve as natural reservoirs, shedding the virus in their urine, saliva, and feces^3^. There is no specific treatment for hantavirus, only supportive care. Prevention and control hinge on avoiding contact with rodents and their excretions, a critical measure for rural workers^4^. HCPS presents as an acute febrile illness with severe cardiopulmonary compromise. Initial symptoms—including fever, headache, and body aches—are non-specific, making early diagnosis difficult^5,6^. The incubation period ranges from three to 60 days, with most cases developing symptoms around two weeks after exposure^7^.

In the last decade, hantavirus cases have increased in Brazilian Southern, Southeastern, and Central-Western states. Identifying the specific hantavirus responsible for infections is crucial as multiple viral lineages with distinct virulence profiles may coexist across broad geographic areas. In Brazil, the Araraquara virus has been consistently linked to severe HCPS and high lethality^8^. Although, the host range has recently expanded, with the identification of the virus in the Atlantic Forest^9^, and Buritiense virus in the Amazon and Cerrado biomes^10^.

This rise is linked to agricultural expansion and population growth, which disrupt ecosystems and favor outbreaks^3,11^. The epidemiology is complex, with transmission also tied to rodent population dynamics and environmental factors like deforestation and construction projects^2^. The Brazilian epidemiological surveillance aims toward the early detection of cases and outbreaks of this compulsory and immediate notifiable disease^2,12^. According to the Brazilian Ministry of Health, the Brazilian South accounts for 35.4% of all cases. The Santa Catarina State reports the highest number of the 2,360 confirmed cases from 1992 to 2023^13^. Thus, this study aims to describe the epidemiological profile of hantavirus cases in Santa Catarina from 2009 to 2019 based on Ministry of Health data.

## Materials and Methods

The study population consisted of all positive hantavirus cases that were reported to the Brazilian National System for Notifiable Diseases (SINAN) from January 1 2009 to December 31, 2019. The scope of this study was limited to the Santa Catarina State, which is composed of 295 municipalities in seven geographic regions, with a total estimated population of 7,125,502 inhabitants^14^.

**Figure 1:**
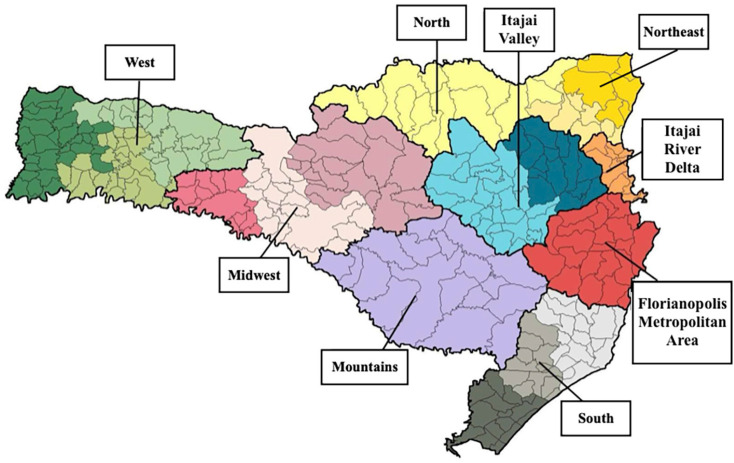
Macroregions according to the State Department of Health of the Santa Catarina State, Brazil.

For statistical analysis, a descriptive case series study was conducted to evaluate the evolution of positive cases, and a retrospective longitudinal study was carried out to assess the outcome (death) of positive hantavirus cases in Santa Catarina in the specified period. The following variables were analyzed: (i) Person: sex, age group, education attainment, and residence zone. (ii) Time: date of symptom onset. (iii) Place: municipality of residence (Table 1) and health region (Figure 1). (iv) Epidemiological history: exposure situations and infection environment. (v) Clinical data: signs and symptoms, laboratory results, and therapeutic procedures.

**Table 1:** Number of cases of hantavirus per municipality in Santa Catarina State between 2009 and 2019

City	Year											
	2009	2010	2011	2012	2013	2014	2015	2016	2017	2018	2019	Total
Alfredo Wagner	0	1	0	0	0	0	0	0	0	0	0	1
Angelina	0	1	0	0	0	0	0	0	0	0	0	1
Anitapolis	0	0	0	0	0	0	0	1	0	0	0	1
Antonio Carlos	0	0	0	0	0	0	1	0	0	0	0	1
Arroio Trinta	1	1	0	0	0	0	0	0	0	0	0	2
Aurora	0	0	0	0	0	0	0	1	0	0	0	1
Balneario Piçarras	1	0	0	0	0	0	0	0	0	0	0	1
Benedito Novo	0	1	0	0	0	0	0	0	0	0	0	1
Blumenau	0	1	0	0	0	0	0	0	0	0	0	1
Bom Jardim da Serra	0	0	0	0	0	0	1	0	0	0	0	1
Bom Retiro	0	0	0	1	0	0	1	0	0	0	0	2
Cacador	0	1	0	0	0	0	1	0	0	0	1	3
Campos Novos	0	0	0	0	0	0	0	0	0	0	4	4
Campo Alegre	1	0	0	0	0	1	1	0	0	0	0	3
Canelinha	1	0	0	0	0	0	0	0	0	0	0	1
Canoinhas	0	0	0	1	0	0	0	1	0	0	0	2
Capinzal	1	0	2	0	0	0	0	0	0	0	0	3
Catanduvas	0	0	0	0	0	0	1	0	0	0	0	1
Chapeco	0	0	2	2	0	1	2	0	0	0	2	9
Concordia	1	2	0	1	1	0	0	1	2	0	3	11
Cunha Pora	1	0	0	0	0	0	0	0	0	0	0	1
Descanso	0	0	0	0	0	0	1	1	0	0	0	2
Frei Rogerio	0	0	0	0	0	0	0	0	0	0	1	1
Florianopolis	0	0	0	0	0	1	0	0	0	0	1	2
Grao Para	1	0	0	0	0	0	0	0	0	0	0	1
Guabiruba	0	0	0	0	0	2	0	0	0	0	0	2
Guaraciaba	0	0	0	0	1	0	1	0	0	0	0	2
Guaramirim	0	0	0	0	0	0	0	1	0	0	0	1
Imarui	0	0	0	0	1	0	0	0	0	0	0	1
Imbituba	0	0	0	0	1	0	0	0	0	0	0	1
Iomere	0	0	0	0	1	0	0	0	0	0	0	1
Ipira	0	0	0	0	0	0	1	0	0	0	0	1
Ipora do Oeste	0	0	1	1	0	0	0	0	0	0	0	2
Ipuaçu	0	0	0	0	1	0	0	0	0	0	0	1
Ipumirim	1	1	0	2	0	1	0	0	0	0	0	5
Iraceminha	0	1	0	0	0	0	0	0	0	0	0	1
Irineopolis	0	0	0	0	0	0	0	1	0	0	0	1
Itaiopolis	0	0	0	2	1	0	0	0	0	0	0	3
Itapiranga	0	1	0	0	0	0	0	0	0	0	0	1
Ituporanga	0	0	1	0	0	0	0	0	0	0	1	2
Jabora	0	0	1	0	0	2	0	0	0	0	0	3
Jaragua do Sul	0	0	0	0	1	0	0	0	0	0	0	1
Joacaba	0	0	0	1	0	1	0	0	0	0	0	2
Joinville	0	0	1	1	0	0	0	0	0	0	0	2
Lages	0	0	0	2	0	0	0	0	0	0	0	2
Laurentino	0	1	0	0	0	0	0	0	0	0	0	1
Leoberto Leal	0	1	0	0	0	0	0	0	0	0	0	1
Lindoia do Sul	0	0	0	0	0	1	0	0	0	0	0	1
Lontras	0	0	0	0	0	0	0	0	1	0	0	1
Luzerna	0	0	0	0	0	1	0	0	0	0	0	1
Mafra	0	0	0	0	0	0	1	0	0	0	0	1
Matos Costa	0	0	0	0	0	0	0	0	1	0	0	1
Mirim Doce	0	1	0	0	0	0	0	0	0	1	0	2
Monte Carlo	0	0	0	0	0	0	0	1	0	0	0	1
Nova Trento	0	0	0	2	0	0	0	0	0	0	0	2
Orleans	0	0	2	0	0	0	0	0	0	0	0	2
Otacilio Costa	0	0	0	0	0	1	0	0	0	0	0	1
Palma Sola	0	1	0	0	0	0	0	0	0	0	0	1
Palmitos	1	0	1	0	0	0	0	0	0	0	0	2
Papanduva	0	0	0	1	0	0	0	0	0	0	0	1
Peritiba	0	0	0	0	1	1	0	0	0	0	0	2
Pinheiro Preto	0	0	0	0	0	0	1	0	0	1	0	2
Ponte Alta do Norte	0	0	0	0	0	0	0	0	1	0	0	1
Porto Uniao	0	0	0	0	3	0	0	0	0	0	0	3
Pouso Redondo	0	0	1	0	1	0	0	0	0	0	0	2
Presidente Castello Branco	0	0	0	0	0	0	0	0	0	1	0	1
Presidente Getulio	0	0	0	0	1	0	0	0	0	0	0	1
Presidente Nereu	0	0	0	0	0	1	0	0	0	0	0	1
Quilombo	0	1	0	0	0	0	0	0	0	0	0	1
Rancho Queimado	0	0	0	0	1	0	0	1	0	0	0	2
Rio do Oeste	0	1	0	0	0	0	0	0	0	1	0	2
Rio do Sul	1	0	1	0	0	0	0	0	1	0	0	3
Rio Fortuna	0	0	1	1	0	0	0	0	0	0	0	2
Rio Negrinho	0	0	1	0	2	0	0	0	0	0	0	3
Riqueza	1	0	0	0	0	0	0	1	1	0	0	3
Santa Terezinha	0	0	0	0	0	0	1	0	0	0	0	1
Santo Amaro da Imperatriz	0	0	1	0	0	0	0	0	0	0	0	1
Sao Bonifacio	0	1	0	0	4	0	0	0	0	0	0	5
Sao Cristovao do Sul	0	0	0	0	0	0	0	0	1	0	0	1
Sao Joao Batista	0	0	0	0	1	0	0	0	0	0	0	1
Sao Jose	0	0	1	0	0	0	0	0	0	0	0	1
Sao Jose do Cedro	0	0	0	0	0	1	0	0	0	0	0	1
Sao Jose do Cerrito	0	0	1	0	0	0	1	0	0	0	0	2
Sao Ludgero	0	0	0	0	0	1	0	0	0	0	0	1
Seara	0	1	0	0	2	0	0	0	1	0	0	4
Serra Alta	0	0	0	0	0	0	1	0	0	0	0	1
Taio	0	0	1	0	0	0	0	0	0	0	0	1
Tangara	0	0	0	0	0	0	1	1	0	0	0	2
Timbo	0	1	0	0	0	0	0	0	0	0	0	1
Timbo Grande	0	0	0	0	0	0	0	0	1	0	0	1
Uniao do Oeste	0	0	0	0	0	0	0	0	0	1	0	1
Urussanga	0	0	0	0	1	0	0	0	0	0	0	1
Videira	1	0	1	0	0	0	0	0	0	0	0	2
Vitor Meireles	1	0	0	0	0	0	0	0	0	0	0	1
Xanxere	0	1	0	0	0	0	1	0	0	0	1	3
Xavantina	0	0	0	0	0	0	0	0	2	0	0	2
Xaxim	0	0	0	0	1	0	0	2	0	0	0	3
Total	14	21	20	18	26	16	18	13	12	5	14	177

For the descriptive study, central tendency, proportion, lethality, and case density analyses were performed on TABWIN (version 4.15, DataSUS, Brasilia, DF, Brazil), Microsoft Office Excel, and MapInfo (version 2023.x, Precisely, Burlington, MA, USA). For the retrospective longitudinal study, median, mean, standard deviation, coefficient of variation, and range analyses were performed on Symbolab (version 12.1.0, Learneo, Inc. business, Redwood City, CA, USA).

The data were obtained from the SINAN database in June of 2021. It compiles the mandatory notification/investigation forms for suspected or confirmed cases of diseases, illnesses, or public health events that are reported by public and private health facilities, as per Consolidation Ordinance no. 4/GM/MS of September 28, 2017^15^.

### Ethics

The used data are shown collectively, without mentioning names or harming the involved individuals. Access to the SINAN database was requested from the Epidemiological Surveillance Directorate via the Access to Information Law Nº 2,527 of November 8, 2011. Following the approval criteria of the Santa Catarina State Department of Health, this project was submitted to the Research Ethics Committee (DIVE/SC) for authorization and commitment to data use. The project received official approval on November 19, 2019. This study was exempted from analysis by the Human Research Ethics Committee of our institution, in accordance with Resolution 510/2016.

## Results

**Figure 2:**
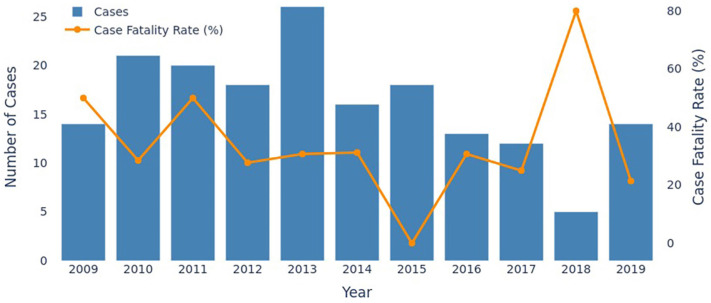
Annual distribution of hantavirus cases in Santa Catarina State by year of occurrence, 2009 to 2019 with the case fatality rate (CFR).

A total of 3,623 suspected hantavirus cases were reported to the SINAN database in Santa Catarina from January 1 2009 to December 31, 2019. Of these, this study included the 177 confirmed ones. The annual distribution of cases peaked in 2013, followed by a significant decline, reaching the lowest number in 2018 before increasing again in 2019 (Figure 2).

**Figure 3:**
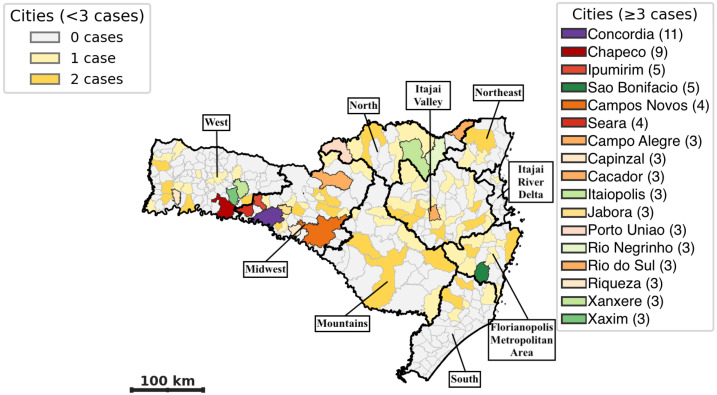
Geographic distribution of positive notifications of hantavirus in Santa Catarina State from 2009 to 2019. Highlighted cities with more than three cases by region: West: Chapeco, Riqueza, Xanxere and Хахіm. Midwest: Concordia, Ipumirim, Campos Novos, Seara, Capinzal, Cacador, Jabora. Florianopolis metropolitan area: Sao Bonifacio. North: Campo Alegre, Itaiopolis, Porto Uniao, Rio Negrinho. Itajai Valley: Rio do Sul.

All Santa Catarina State regions reported cases (Table 2). The highest concentration of cases (57.6%) occurred in the West, Midwest, and Mountain regions, followed by the Itajai Valley and the Florianopolis metropolitan area. The lowest number of cases occurred in the Itajai River Delta and Southern regions. In total, 96 of the 295 municipalities in the state registered hantavirus. The municipalities with the highest number of cases were Concordia (11), Chapeco (9), Ipumirim (5), and Sao Bonifacio (5), followed by Campos Novos (4) and Seara (4). The remaining municipalities had between one and three cases (Figure 3).

**Table 2:** Number and percentage of cases by health region, according to the patient's municipality of residence, 2009 to 2019

Area	Cases	%	Lethal cases	%
West	35	19.8	3	8.5
Midwest and Mountains	67	37.9	21	31.3
North and Northeast	18	10.2	8	44.4
Itajai Valley	28	15.8	15	53.5
Itajai River Delta	1	0.5	-	-
Florianopolis Metropolitan	21	11.8	6	28.5
South	7	4.0	2	28.5
Total	177	100	55	31.7

### Sociodemographic and epidemiological profile

Most confirmed cases (77.4%) were male (n=137), while females accounted for 22.6% (n=40). The most affected age group was aged between 20 to 49 years, with a mean age of 38 years. Patients aged under 14 years (n=7) and over 60 years (n=10) represented a combined 5.6% of cases.

Regarding educational background, 17% of the notification forms lacked this information. Thus, this analysis used 146 cases. Most patients (64.5%) had completed or incomplete primary education, whereas 25.3% had completed secondary education. The predominant residential area was rural, accounting for 58% of the cases. This study considered 10 risk situations for hantavirus infections; the most common were cleaning or similar activities (n=112), followed by deforestation and agricultural activities (n=92), and direct contact with rodents (n=76).

### Clinical characteristics and outcome

Of the 177 cases, patients reported 18 symptoms, the most frequent of which were fever (93%), headache (81%), myalgia (72%), nausea/emesis (69%), and dyspnea (67%). Other common symptoms included abdominal pain (55%), cough (53%), respiratory failure (48%), dizziness/vertigo (48%), and chest pain (45%). Less frequent symptoms included heart failure (n=12) and hemorrhagic manifestations and asthenia (n=3 each).

Laboratory data were available for a portion of the cases. Serological tests, hemograms, and chest X-rays were performed in 94.1% (n=162/172) of patients. Serology was reactive in 94.6% (n=160/169) of cases; in four patients no sample was collected, in other four with non-reactive results and one with an inconclusive result were confirmed by clinical-epidemiological criteria. Immunohistochemistry and polymerase chain reaction assessed the disease in nine and seven patients, respectively, all with positive results. Of the hemograms with available data (76.4%, n=135/177), 53.9% (n=75/139) showed altered hematocrit (>45%), 54.5% (n=72/132) had increased urea and creatinine and 53.8% (n=71/132) had thrombocytopenia. Elevated aspartate transaminase (63.3%, n=88/139) and alanine aminotransferase (60.4%, n=84/139) also occurred commonly, whereas reactive lymphocytes only emerged in 18.4% (n=24/130) of cases. Chest X-rays were recorded on 70.6% (n=125/177) of forms, evincing diffuse pulmonary infiltrates in 62.4% (n=78/125), localized infiltrates in 10.5% (n=13/123), and pleural effusion in 13% (n=16/123) of patients.

Hospitalization was required for 89.7% (n=157/175) of positive cases. The fourth quarter of the year (Sep-Dec) showed the highest hospitalization rate (34.1%, n=53/155). The most common therapeutic support used antibiotics (85.2%, n=133/156), followed by mechanical ventilation (40.5% n=64/158) and continuous/bilevel positive airway pressure (12%, n=18/149). Ribavirin was used in only 5.2% of hospitalized cases, whereas vasoactive drugs were used in 28.3% (n=42/148) and corticosteroids in 25.6% (n=38/148).

The case fatality rate (CFR) for the study period totaled 33.3% (n=59/177), with 68.9% (n=122/177) of cases evolving to cure. Hantavirus cardiopulmonary syndrome was the predominant clinical form (58.7%, n=104/177), whereas the prodromal or non-specific form occurred in 41.2% (n=73/177) of cases. The annual CFR varied from 21.4% in 2019 to 80%, with 2015 having the lowest and 2018 the highest lethality rate.

### Clinical course and lethality analysis

The mean time from symptom onset to first care totaled 3.5 days (±2.6). For hospitalized patients, this period equaled 4.5 days (±3.0). The mean time from symptom onset to medical discharge for cured patients totaled 8.3 days (±6.7), whereas it equaled 6.4 days (±4.8) for those who died. Average hospital stay was 4.8 days (±7.1) for patients who recovered, compared to 3.3 days (±12.7) for those who died.

The CFR was similar between urban (31.5%) and rural (31%) residents. By geographic area, the highest lethality occurred in the Itajai Valley (53.5%, n=15/28), followed by the Northern-Northeastern region (44.4%, n=8/18). The lowest CFR occurred at the West (8.5%, n=3/35). Other areas ranged from 28.5 to 31.3%. The Itajai Delta area had only one non-fatal case.

### Lethality by demographic and clinical factors

Over the study period, males (32.1%, n=44/137) had a higher CFR than females (27.5%, n=11/40). The highest CFR by age group was in adolescents aged 15-19 (50%, n=4/8) years, followed by patients aged from 50 to 64 years (34.7%, n=8/32). The lowest rate occurred in patients aged older than 65 years (11.1%, n=1/9). Most deaths occurred in patients with incomplete primary (28 of 94) and secondary education (11 of 37), both with a CFR of 29.7%.

An analysis of CFR by epidemiological risk factors showed the highest lethality in patients exposed during general cargo transport activities (40%, n=8/20), followed by those who slept in tents, sheds, or barns (32.2%, n=10/31). CFRs were similar among patients with reported direct rodent contact, deforestation, cleaning activities, or grain handling and storage, from 28.9 to 24.1%.

The lethality rate totaled 43.5% in patients who presented with or developed respiratory symptoms (51 of 117) in comparison with the ones without them (6.8%, n=4/58). In total, two cases lacked such information. CFR was also higher in patients who required mechanical ventilation (65.6%, n=42/64) than in those who had no such need (13.8%, n=13/94), had elevated hematocrit (45.3% - 34 of 75) when compared with those with normal values (16.6%, n=9/54), and showed diffuse or localized pulmonary infiltrates on X-ray (51.2%, n=40/78) in comparison to those with normal X-ray results (12.7%, n=6/47). In total, 52 cases lacked information on X-ray patterns; the patient died in nine of these cases (31.5%).

Finally, lethality was higher (36%, n=35/97) in patients who sought medical care within the first three days of symptom onset than in those who did so from four to seven days (25%, n=16/64) and from eight to 14 days (25%, n=4/16).

## Discussion

Hantavirus is a severe, acute, re-emerging zoonosis prevalent across the Americas, with confirmed cases reported in in all Brazilian regions. The Southern Brazil has the highest number of human cases^13^. In our cohort, cases peaked in Santa Catarina State in 2013, which was consistent with a national trend, with a rise in positive cases in the Central-West and Southeast regions^16^. These peaks are often linked to natural phenomena such as the synchronized flowering of bamboo (*Poaceae: Bambusoideae*). This is common in Southern Brazil and provides a plentiful food source, leading to an exponential increase in the rodent population—the natural hosts of hantavirus^3^. These findings are in the line with the expected five-to-seven-year intervals between flowering events, with the last one occurring in 2011 and 2012^3^. The seasonality of cases, based on symptom onset, showed a higher incidence in October, November, and December.

According to Oliveira *et al*.^17^, the increase in cases in the West, Midwest, and Mountain regions of Santa Catarina is associated with the predominance of Atlantic Forest and Araucaria Forests, which favor the maintenance and proliferation of wild rodents. Regions with agricultural economic profiles and expanding urban areas are more susceptible to the spread of emerging diseases like hantavirus^18-20^. Maize cultivation, common in these regions, is a significant risk factor for HCPS transmission^21^.

The sociodemographic profile of hantavirus cases in Santa Catarina State was consistent with national data, in which the typical patient is a male young adult of working age with low educational attainment who lives in rural areas^16,22-24^. The epidemiological risk factors in this study is in line with previous national studies, which have highlighted occupational activities as the primary source of hantavirus infection^1,3^. The similar percentages for "probable infection environment" between "home" and "work" can be attributed to reporting complexities as many rural residents live and work in the same location^25,26^.

The clinical presentation of hantavirus is non-specific, similar to other viral diseases^23,27,28^; although, predominantly characterized by fever, headache, and myalgia. In areas in which dengue and hantavirus cases overlap, early serological testing for both infections—such as NS1 or IgM for dengue and ELISAIgM for hantavirus—should be promptly performed to enable differential diagnosis, guide appropriate patient management, and improve outcomes^29^.

This clinical resemblance and the acute progression of the disease make differential diagnosis difficult, hindering specific therapeutic intervention^2,20,30^. Laboratory results from this study are consistent with classic hantavirus cases^27,31^ The 89.7% found hospitalization rate mirrors the national scenario^31^. The higher hospitalization rate during the fourth quarter is presumed due to warmer temperatures from September to December, which increase outdoor activities and, consequently, the risk of exposure^30^.

Despite the high incidence of hantavirus in Santa Catarina, its overall lethality rate is below the national average of 42%^23^. Our data showed an increased interval from symptom onset to outcome severity, highlighting that early care is crucial for a favorable prognosis. But, on the other hand, lethality was higher in patients who sought medical care within the first three days of symptom onset. Symptom-onset data were based on patient self-reports during the initial clinical encounter and then input into the Ministry of Health surveillance system. Although early HCPS symptoms are often nonspecific, these records are considered reliable as they constitute the standard reference for national reporting despite the possibility of minor underestimation.

CFR was nearly identical between urban (31.5%) and rural (31%) residents. However, as the population density in Santa Catarina State is 5.7 times higher in urban areas than rural ones^14^, this finding underscores the disproportionately high risk for rural residents.

Consistent with other studies^27,32,33^, CFR was higher in patients requiring mechanical ventilation, those with elevated hematocrit, and those with diffuse or localized pulmonary infiltrates on chest X-ray. According to Fonseca *et al*.^16^, high lethality, even with early care, can be linked to the severity of the clinical presentation, with signs such as shock, hypotension, and respiratory distress indicating rapid progression. The fact that the overall lethality in Santa Catarina State is below the national average may be related to the efficiency of its epidemiological surveillance and medical teams in recognizing the clinical picture and adopting appropriate therapeutic measures early.

### Limitations

A limitation of this study refers to the period of the collected data. This research defined such temporal scope with the critical function of creating a baseline to avoid confounding the reported findings with the substantial and often overwhelming effects of the COVID-19 pandemic. However, this intentional exclusion constitutes a primary temporal limitation as the results may fail to fully reflect post-2019 changes in epidemiological patterns, health system utilization, or surveillance practices that could significantly impact the studied phenomena. A separate but equally important limitation stems from the reliance of this study on the SINAN database. While it is a crucial national resource compiling mandatory notification/investigation forms, its integrity depends on the completeness of reporting from public and private health facilities. The analysis in this study was restricted to available fields. Incomplete case reporting, particularly missing data, is an inherent constraint of large-scale passive surveillance systems. Therefore, the observed associations and incidence rates may constitute an underestimation. Moreover, the lack of comprehensive information for every reported case limits the depth of the descriptive analysis in this research.

## Conclusion

The hantavirus profile in Santa Catarina State aligns itself with the national scenario but also shows significant regional differences that necessitate tailored surveillance and prevention measures. There exists an urgent need for continuous education and training for healthcare professionals in public and private sectors to improve early diagnosis and clinical management, especially in regions with low reported cases (Itajai river delta and South) that may show underreporting.

The high lethality associated with agricultural activities reinforces the need for a multisectoral surveillance system involving health, agriculture, and environmental agencies. Developing a unified protocol that accounts for the socio-demographic and economic realities of Santa Catarina State could improve prevention and control strategies, particularly for occupational cases.

To reduce lethality and ensure timely therapeutic support, medical teams must be more vigilant in their differential diagnosis and clinical management from the first patient contact onward. A standardized protocol should require follow-up appointments for patients with non-specific hantavirus-compatible symptoms, especially for adolescents aged 15-19 years and in regions with low notification rates.

This study shows the value of using a governmental epidemiological database to find key patterns and inform public health strategies. However, reporting gaps and inconsistencies limit data usefulness. Public information and awareness campaigns are also essential to educate the population on disease prevention as this remains the foundation of effective health surveillance. Hantavirus infection continues to be a neglected disease in Brazil, and this study contributes to raising awareness and disseminating knowledge among healthcare providers.

## Data Availability

The authors will make no additional data available due to ethical restrictions related to patient confidentiality.
